# Role of Laparoscopy in Diagnosing and Treating Acute Nonspecific Abdominal Pain

**DOI:** 10.7759/cureus.18741

**Published:** 2021-10-13

**Authors:** Barza Afzal, Shabbar H Changazi, Zulqarnain Hyidar, Sumera Siddique, Aveena Rehman, Samiullah Bhatti, Qamar Ashfaq Ahmad, Muhammad Waris Farooka

**Affiliations:** 1 General Surgery, Services Hospital Lahore, Lahore, PAK; 2 Surgical Special Unit, Services Hospital Lahore, Lahore, PAK; 3 Surgical Unit 2, Services Hospital Lahore, Lahore, PAK; 4 Surgery, Services Hospital Lahore, Lahore, PAK; 5 Department of Surgery, Services Institute of Medical Services, Lahore, PAK

**Keywords:** treatment, diagnosis, non-specific abdominal pain, emergency laparotomy, emergency laparoscopy

## Abstract

Background

Nonspecific abdominal pain (NSAP) is a pain for which no immediate cause is evident on acute admission and does not necessitate emergency surgical intervention. NSAP is a frequent reason for presentation in the emergency department (ED). Laparoscopy is a well-established technique that allows a surgeon to visualize the abdominal cavity after insufflation through a few small incision ports. Despite the increasing availability of laparoscopic investigation, the availability of a laparoscope in the ED settings in Pakistan is low due to the expense and maintenance needs of the system.

Objective

This study aimed to evaluate the role of laparoscopy in diagnosing the cause of acute NSAP and its role in treating the pathology of disease in patients presenting to the emergency department (ED) of Services Hospital, which is a government sector hospital in Lahore, Pakistan.

Materials and methods

This study was conducted in Services Hospital Lahore, Pakistan, from January 1, 2016 to December 31, 2019. The study included patients aged 12 to 70 years of either sex who presented to the ED with abdominal pain for whom no diagnosis could be achieved on clinical assessment, laboratory findings, and radiological findings (x-ray abdomen, ultrasonography, and computed tomography scan). All study participants underwent diagnostic laparoscopy under general anesthesia. Patients were monitored weekly via follow-up postoperatively for the first month and then monthly for 12 months. All study data were recorded on a predesigned proforma. The data were analyzed using IBM Corp. Released 2012. IBM SPSS Statistics for Windows, Version 21.0. Armonk, NY: IBM Corp.

Results

A total of 122 patients diagnosed with acute NSAP were enrolled in our study (mean age, 46.4 ± 20.3 years). The study population consisted of 52 male patients (42.6%) and 70 female patients (57.4%). Our study participants had a mean body mass index of 24.2 ± 3.3 kg/m^2^. The most common ED presentation was lower abdominal pain. One hundred sixteen patients (95.1%) had positive findings on laparoscopy, while six patients (4.9%) had no identified pathology on laparoscopy. The most frequent pathology was appendicular in origin, followed by pelvic inflammatory disease. Surgical management of patients through laparoscopy was performed in 97 patients (79.5%). Conversion to laparotomy was done in 12 patients (9.8%). Definite diagnosis was established in 118 patients (96.7%). Port site infection occurred in four patients (3.3%), chest infection in five patients (4.1%), deep venous thrombosis in one patient (0.8%), and anastomotic leakage in one (0.8%) patient. Four patients (3.3%) developed recurrence of symptoms.

Conclusions

This study explored the role of laparoscopy in diagnosing and treating patients presenting to the ED with acute NSAP. According to our results, laparoscopy is a safe and effective method for diagnosing and treating acute NSAP with low postoperative morbidity and potentially decreased risk of postoperative complications. Physicians should consider laparoscopy as a first-line invasive investigation for patients presenting with undiagnosed acute abdominal pain.

## Introduction

Non-specific abdominal pain (NSAP) is pain for which no immediate cause can be found on admission and does not necessitate emergent surgical intervention. NSAP is a frequent reason for presentation in surgical emergencies, and it may be caused by various conditions such as appendicitis, ectopic pregnancy, pelvic inflammatory disease (PID), torsion of adnexa, cholecystitis, and pancreatitis. Diagnoses may be hindered by a variety of factors, leading to poor patient outcomes [1,2].

Relatively young female patients comprise the largest subgroup of NSAP patients. These patients often have repeated episodes, are difficult to discharge, and undergo a battery of tests. NSAP frequently mimics appendicitis, and some patients undergo an appendectomy that ultimately reveals a healthy appendix. This leads to increased financial costs and burdens for both the patient and the healthcare facility. The risk of unnecessary surgery against a missed pathology complicates the surgeon’s decisions [3].

Laparoscopy is a well-established technique that visualizes the abdominal cavity after insufflation via ports inserted through small incisions. This allows for the introduction of the laparoscope and other instruments for visualization and intervention. Many advanced surgeries are now performed via this approach [4].

However, a certain level of surgical expertise in laparoscopy is essential, especially in an emergency setting. Despite the increasing availability of laparoscopic technology in many institutions, it is still relatively rare in Pakistan given the costs associated with setup, maintenance, and trained personnel in the emergency department (ED), which is often separate from the main operation theater [5,6]. We conducted this study to evaluate the role of laparoscopy in diagnosing the causes of NSAP and treating the pathology of the disease in patients presenting to the ED.

## Materials and methods

This study was conducted in the Surgical Department at the Services Hospital in Lahore, Pakistan, from January 1, 2016 to December 31, 2019. The hospital institutional review board approved the study design. We computed a sample size of 122 patients at a 95% confidence level with a 5% margin of error. We used 13% as our expected percentage of NSAP prevalence. The study included patients aged 12 to 70 years of either sex who presented with abdominal pain for which diagnosis could not be made via clinical assessment, laboratory evaluations, and radiological findings (including ultrasonography and computed tomography). We excluded patients who had definitive diagnosis on laboratory investigations (such as elevated serum amylase and lipase levels) or on radiological investigations (such as free air under the diaphragm on x-ray, inflammed gallbladder/ascites on ultrasonology or inflammed appendix, or sigmoid colon on computed tomography). Patients with hemodynamic instability (i.e., blood pressure < 90/50 mmHg) and/or American Society of Anesthesiologist type 4 were also excluded from the study.

Patients who met the inclusion criteria were admitted through the surgical ED at Services Hospital in Lahore. All patients provided informed consent and underwent diagnostic laparoscopy under general anesthesia. Patients were counseled on the possibility of conversion of laparoscopic surgery to laparotomy depending on perioperative findings. If necessary, an incision for open surgery was made. All laparoscopic findings were authenticated and substantiated via cytology, histopathology, culture sensitivity, and specific blood tests or ascitic fluid tests. Patients were monitored via follow-up weekly for the first month postoperatively and then monthly for 12 months. All the data were recorded on a predesigned proforma.

Data was entered and analyzed using IBM Corp. Released 2012. IBM SPSS Statistics for Windows, Version 21.0. Armonk, NY: IBM Corp. The quantitative variables such as age and body mass index (BMI) were presented by calculating mean and standard deviation. The qualitative variables such as sex, cause of NSAP, and type of procedure performed were presented as frequencies and percentages.

## Results

The study enrolled 122 patients diagnosed with acute NSAP (mean age, 46.4 ± 20.3 years). The study population consisted of 52 male patients (42.6%) and 70 female patients (57.4%). Our study participants had a mean BMI of 24.2 ± 3.3 kg/m2. The most common presentation was lower abdominal pain. The mode of presentation for all patients is listed in Table 1.

**Table 1:** Presenting symptoms of NSAP patients Abbreviation: NSAP: nonspecific abdominal pain.

Symptoms		N (%)
Site of pain	Generalized	14 (11.5%)
	Right hemi-abdomen	4 (3.3%)
	Left hemi-abdomen	17 (13.9%)
	Upper abdomen	4 (3.3%)
	Lower abdomen	83 (68.0%)
Associated Features	Fever	30 (24.6%)
	Nausea and vomiting	98 (80.3%)
	Anorexia	40 (32.8%)

We noted positive findings on laparoscopy in 116 patients (95.08%), and in six patients (4.92%), no pathology was identifiable via laparoscopy.

A majority of patients (n=97; 79.2%) received laparoscopy surgical management. Twelve patients (9.8%) had to convert from laparoscopic surgery to laparotomy. The remaining 13 patients (10.66%) were managed conservatively with intravenous antibiotics and analgesics. The pathological findings and respective treatments are presented in Table 2.

**Table 2:** Laparoscopy diagnostic results and treatments Abbreviations: PID: pelvic inflammatory disease; IV: intravenous; TAPP: transabdominal preperitoneal patch; NA: not applicable.

Finding/Diagnosis on Laparoscopy	Laparoscopic Treatment	Alternative Treatment	N (%)
Appendicular pathology	Appendectomy	NA	55 (45.1%)
PID	Drainage of puss and washing	NA	21 (17.2%)
Matted gut with mild ascites	Aspiration of ascitic fluid and omental biopsy	NA	10 (8.2%)
Multiple ileal strictures	Converted to laparotomy	Resection of segment and anastomosis/ Stricturoplasty	6 (4.9%)
No finding	Managed conservatively	Managed with IV antibiotics and analgesics	6 (4.9%)
Ovarian torsion	Detorsion and fixation	NA	4 (3.3%)
Gut ischemia	Converted to laparotomy	Resection of effected segment	4 (3.3%)
Sigmoid colon diverticulitis	None	Managed with IV antibiotics	3 (2.5%)
Enlarged mesenteric lymph nodes	Lymph node biopsy	NA	2 (1.6%)
Concealed gall bladder perforation	Cholecystectomy	NA	2 (1.6%)
Salpingitis	None	Managed with IV antibiotics	2 (1.6%)
Meckel's diverticulitis	Converted to laparotomy	Wedge resection	2 (1.6%)
Jejunal diverticulitis	None	Managed with IV antibiotics	2 (1.6%)
Inguinal hernia	TAPP	NA	1 (0.8%)
Endometriosis	Cautery of spots	NA	1 (0.8%)
Femoral hernia	TAPP	NA	1 (0.8%)

One hundred eighteen patients (96.7%) received a definite diagnosis, and four patients (3.28%) had no definite diagnosis. Acute appendicitis was the most frequent diagnosis, followed by PID and abdominal tuberculosis (Table 3).

**Table 3:** Final diagnoses Abbreviation: PID: pelvic inflammatory disease.

Finding/Diagnosis	N (%)
Appendicular Pathology	50 (40.9%)
PID	21 (17.2%)
Abdominal tuberculosis	17 (13.9%)
Ovarian torsion	4 (3.3%)
Gut ischemia	4 (3.3%)
Sigmoid colon diverticulitis	3 (2.5%)
Lymphoma	3 (2.5%)
Salpingitis	2 (1.6%)
Carcinoid of appendix	3 (2.5%)
Acute cholecystitis/gangrenous gallbladder	2 (1.6%)
Meckel's diverticulitis	2 (1.6%)
Jejunal diverticulitis	2 (1.6%)
Irritable bowel disease	2 (1.6%)
Inguinal hernia	1 (0.8%)
Endometriosis	1 (0.8%)
Femoral hernia	1 (0.8%)

Four patients (3.3%) developed port site infection, five patients (4.1%) developed pneumonia, one patient (0.8%) developed deep venous thrombosis, and one patient (0.8%) had anastomotic leakage. Four patients (3.3%) had a postoperative recurrence of symptoms.

## Discussion

Vague abdominal pain constitutes 13% to 40% of all surgical emergencies [3] and represents a diagnostic challenge. Despite an extensive workup, a proper diagnosis may not be reached for many patients. This is a considerable problem for both patients and health care facilities, and clinicians. The underlying cause in cases of NSAP can be difficult to uncover, and patients may need to undergo several investigations, including surgical exploration, to determine and properly manage the cause. Diagnostic laparoscopy might play an essential role in these cases, as it enables direct visualization of the abdominal cavity and its viscera. Diagnostic laparoscopy also enables the surgeon to take tissue samples and use them as a therapeutic tool. Visualization, sample collection, and corrective intervention via laparoscopy are achieved with minimal patient morbidity and scars. However, a literature review yields a conflicting message-some studies strongly support the use of laparoscopy in challenging cases of NSAP while others are not as supportive [7,8].

More than half of the patients with NSAP were females (57.38%) in our study, a proportion observed by other studies [4,9]. Furthermore, lower abdominal pain was the most common presenting symptom, which is consistent with the findings of Ahmad et al. [3]. The higher prevalence of lower abdominal pain in female patients may be due to anatomic differences between the female and male pelvis [3,10].

The underlying cause of NSAP was the appendix in 56% of cases, which falls into the prevalence of appendix-related vague pain reported by other studies (33% to 73%) [9,11]. We found a much lower incidence of PID and tuberculosis than previous reports [12].

In our study, most patients (95.1%) had positive findings on laparoscopy. Ou et al. reported that diagnostic laparoscopy revealed a definitive diagnosis in 76 of 77 cases (98.7%) [13]. Similarly, Ahmad et al. reported a success rate of 87.3% using diagnostic laparoscopy [3]. Our results in context with the existing literature further support laparoscopy in managing acute NSAP.

Our conversion rate to laparotomy was 9.84% of patients receiving laparoscopy. Waclawiczek et al. reported a conversion rate of 2.7% in 172 cases of acute abdomen requiring laparoscopy [14]. Onders et al. reported a low conversion rate to laparotomy, and a recent study from India also reported that laparotomy could be avoided in 96% of similar cases [15,12]. Our conversion rate is higher than the rates reported in the literature in similar studies. This may be due to laparoscopy use still being in the initial stages of development in Pakistan in ED settings. With time, training, and familiarity with emergency laparoscopy, we expect the conversion rate to laparotomy to decline. Secondly, advanced laparoscopic instruments such as endoscopic gastrointestinal staplers and sealing devices are unavailable in our region.

There are certain limitations to this study. Our study was limited because it was a single-center study, which prohibits the broad generalization of our results. Secondly, the study was limited by its relatively small sample size. A multicenter study with a larger sample is required to support our findings further.

## Conclusions

This study explored the role of laparoscopy in diagnosing and treating patients presenting to the ED with acute NSAP. According to our results, laparoscopy is a safe and effective method for diagnosing and treating acute NSAP with low postoperative morbidity and potentially decreased risk of postoperative complications. Furthermore, this study will help inform updates to practices and guidelines regarding adopting better techniques in Pakistan’s local clinical practices. Therefore, physicians and policymakers should consider laparoscopy as a first-line invasive investigation option for undiagnosed NSAP.
